# Left Para-Duodenal Hernia Presenting With Recurrent Abdominal Pain: A Diagnostic Challenge

**DOI:** 10.7759/cureus.67107

**Published:** 2024-08-18

**Authors:** Nixat J Patel, Jagadeeswar Reddy Peddapati, Shachi Barot, Suhas Mareedu, Prathyusha Erepally, Suraj Bhanja

**Affiliations:** 1 Medicine, Government Medical College Surat, Surat, IND; 2 Surgery, Medicine, Osmania Medical College, Hyderabad, IND; 3 Obstetrics and Gynaecology, Dr M.K. Shah Medical College and Research Centre, Surat, IND; 4 Medicine, All American Institute of Medical Sciences, Black River, JAM; 5 Medicine, Prasad Multispecialty Hospital, Hyderabad, IND; 6 Neurosurgery, MGS Super Speciality Hospital, Delhi, IND

**Keywords:** surgical management, landzert's fossa, intestinal obstruction, laparotomy, left para-duodenal hernia

## Abstract

Paraduodenal hernias (PDH) are a type of internal hernia that results from errors in midgut rotation and failure of mesenteric fusion. Internal hernias themselves are a rare presentation that accounts for less than 1% of total hernias, and paraduodenal hernias contribute more than half of internal hernias. Left paraduodenal hernias (LPDH) occur more frequently as compared to right paraduodenal hernias. Left paraduodenal hernias happen when the small intestine bulges out in the left paraduodenal space, which is also called the space of Landzert. This can cause vague abdominal pain and signs of intestinal blockage, which makes diagnosis difficult. We present a case of a 28-year-old male with recurrent abdominal pain for 7 years, diagnosed with LPDH via laparotomy after inconclusive imaging studies, such as the whirlpool sign on USG. Intraoperatively, jejunal loops were found in Landzert's fossa, hindering hernia repair and adhesiolysis. Clinicians must maintain a high index of suspicion for PDH when assessing nonspecific abdominal symptoms to ensure timely diagnosis and management, optimising patient outcomes.

## Introduction

Recurrent upper abdominal pain is a common complaint in outpatient settings, often attributed to various gastrointestinal and intra-abdominal pathologies. Rare paraduodenal hernias (PDH) in young adults are difficult to diagnose, necessitating detailed case reports [1]. PDH is a type of internal hernia in which loops of the small intestine stick out through a birth defect next to the paraduodenal fossa. It represents an uncommon entity, often masquerading as something else when presenting with non-specific symptoms like recurrent abdominal pain and intermittent bowel obstruction [2]. Concurrent intestinal malrotation significantly complicates diagnosis and necessitates high clinical suspicion [3].

This case holds significance due to its exceptionally rare presentation, illustrative complexity, and the successful surgical intervention that yielded positive outcomes [1]. The relatively uncommon recurrence of PDH and intestinal malrotation underscores the importance of highlighting this particular case, as it addresses a significant gap that exists in the current surgical literature [4]. This case report shows a young patient who had unusual abdominal pain and bowel obstruction. It describes the patient's unique clinical features, how the problem was diagnosed, the surgery that was done, and how well they did after the surgery. The goal is to raise awareness of left paraduodenal hernia (LPDH) and intestinal malrotation [1].

The main objective of this case report is to contribute valuable insights for clinical management, primarily aiming to foster a greater understanding of left PDH and intestinal malrotation. It also contributes valuable details and investigations to the existing literature, aiming to improve clinical recognition and management of these rare surgical entities, ultimately enhancing patient care. It details the unique clinical presentation, diagnosis, surgical management, and postoperative course of paraduodenal hernia and associated intestinal malrotation in a young adult. It aims to enhance understanding and awareness of these rare causes of abdominal pain and obstruction in this population.

## Case presentation

A 28-year-old male with no significant past medical history and no previous abdominal surgeries presented with a seven-year history of recurrent upper abdominal pain. The pain was episodic, insidious in onset, and progressively worsening, primarily localized to the left upper quadrant and described as dull aching. Meals made it worse, which caused the patient to skip meals when they were experiencing painful episodes. There were times when the pain became excruciating, accompanied by abdominal distension and bilious vomiting, which brought about momentary relief. The most recent severe episode occurred one month prior to presentation, prompting hospital admission and further investigation.

On examination, the abdomen was soft and non-tender, with no palpable masses, and vital signs were stable. An ultrasound showed that the mesentery was twisting or rotating along its axis. This is called the typical sonographic "whirlpool sign," and it was found in the right paramedian umbilical region. It was connected to vessels and bowel loops.

The contrast-enhanced CT scan showed that the duodeno-jejunal junction was on the right side of the midline. The duodenum and proximal jejunum were also on the right side, while the rest of the jejunum was in the left upper quadrant. This showed that the intestines were partially misaligned, with the midgut volvulus and mild subacute obstruction.

**Figure 1:**
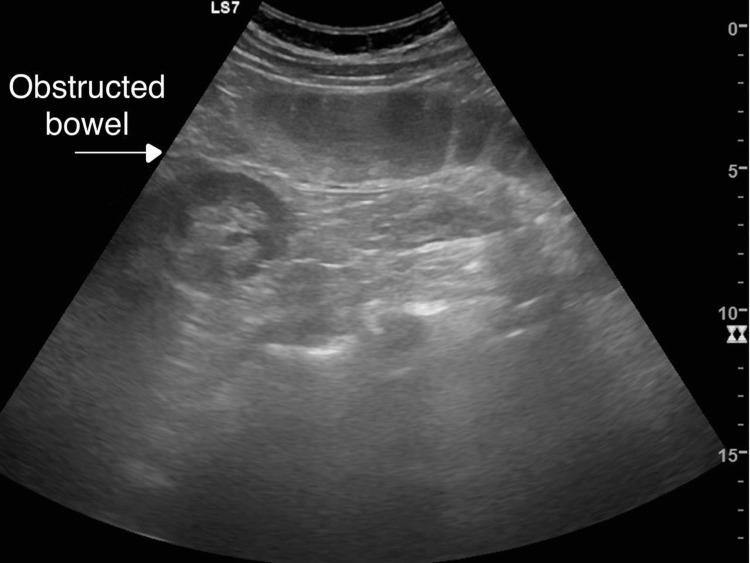
Ultrasound image demonstrating an obstructed bowel.

The ultrasound scan reveals signs of bowel obstruction with distended bowel loops and fluid levels, indicating a blockage in the intestinal tract (Figure 1).

**Figure 2:**
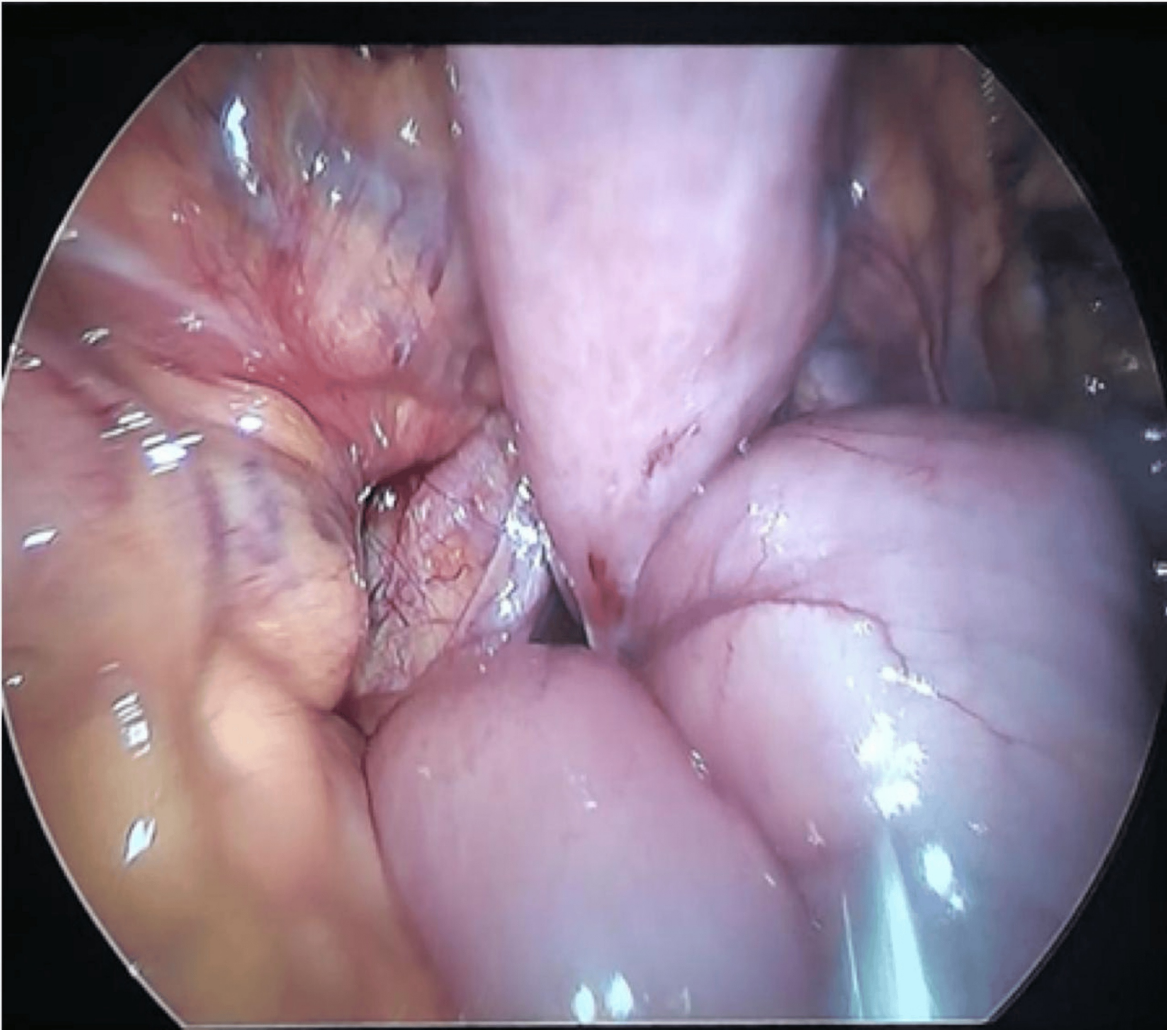
Jejunal loops herniating through the defect, indicating an internal hernia.

**Figure 3:**
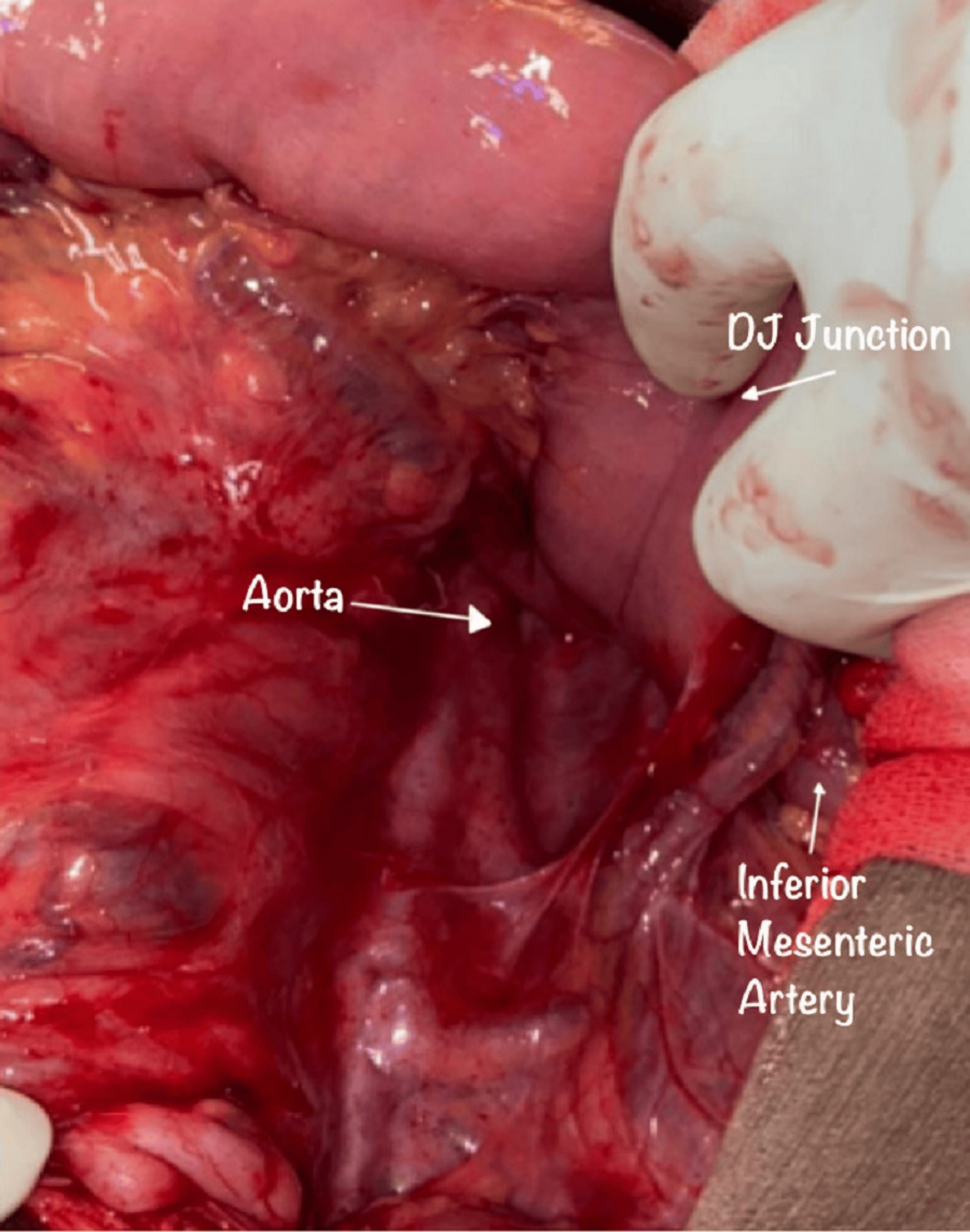
Post-reduction view showing the defect clearly, located at Landzert's fossa.

Intraoperative findings that were consistent with small bowel herniation through the Landzert fossa and led to the diagnosis and confirmation of left paraduodenal hernia (Figures 2, 3).

The patient underwent elective diagnostic laparoscopy and was converted to an exploratory laparotomy, which revealed approximately 30-35 cm of jejunal loops herniating into the Landzert fossa behind the mesocolon, with mesenteric adhesions and engorgement of the jejunal and ileal veins.

The surgical procedure involved the reduction of the herniated bowel loops, obliteration of the herniated sac space, and inter-mesenteric adhesiolysis.

**Figure 4:**
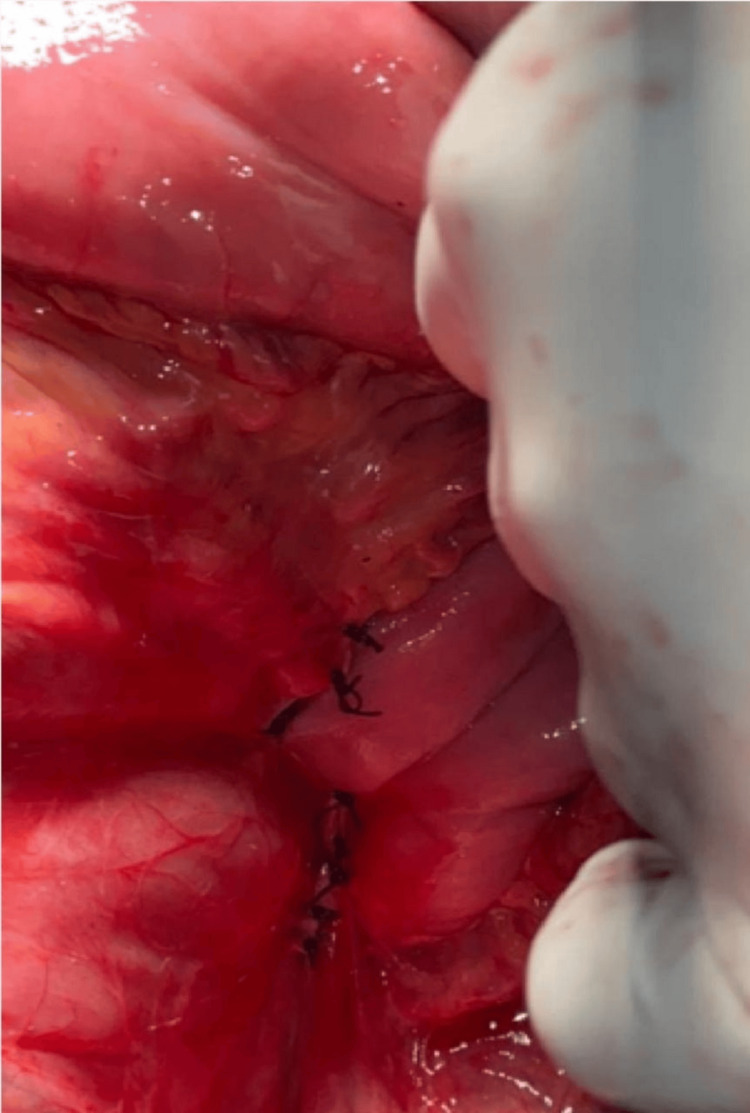
Complete obliteration of Landzert's fossa following the procedure.

Figure 4 demonstrates the obliteration of Landzert's fossa after the surgical procedure, ensuring the hernia defect is closed and the intestines are secured in place.

The postoperative course was uneventful, and the patient was discharged on the fifth postoperative day. Follow-up visits showed complete resolution of symptoms with no recurrence of abdominal pain and no long-term complications, and the patient resumed normal activities.

## Discussion

Left paraduodenal hernias (LPDH) are a rare subtype, representing approximately 30% of all paraduodenal hernias, which in turn account for more than half of all internal hernias. Recent studies have demonstrated a shift in the epidemiology of internal hernias, with postsurgical mesenteric hernias now representing the most common type [5]. This rarity emphasizes the critical importance of reporting and documenting such cases. Detailed case reports contribute significantly to the medical community’s understanding of this uncommon condition, improving diagnostic accuracy and informing better management strategies. Each documented case adds valuable information about presentation, diagnostic pitfalls, and successful therapeutic interventions, which are essential for enhancing patient outcomes.

The majority (53%) of internal hernias are of the rare congenital abnormality known as paraduodenal hernia. They are reported to be more common in men than women, with a ratio of 3:1, and may be detected at any age [6]. Left-sided paraduodenal hernia is more common than right-sided (75%:25%) [7]. Our patient is a classic case of left paraduodenal hernia, which will be the focus of our case study.

In particular, left paraduodenal hernias were caused by the midgut rotating dorsally to the colic branches of the inferior mesenteric artery instead of ventrally. This made the intestines bulge into the mesocolon between the colon and the back wall of the abdomen [8]. Anatomically, many paraduodenal fossae exist. Normally, the mesocolon has to fuse with the posterior peritoneal wall; failure of the fusion results in the formation of the fossa.

When a paraduodenal hernia happens on the left side, it involves a herniation through Landzert's fossa. This type of hernia is different from the more common type that happens on the right side. Because they are located behind the descending colon and close to the inferior mesenteric vein and left colonic vessels; left paraduodenal hernias can make it harder to get to the area for surgery. These anatomical considerations necessitate meticulous surgical planning to avoid vascular injury and ensure successful repair. Compared to right-sided paraduodenal hernias, left-sided hernias often require more intricate dissection and a tailored surgical approach, which can impact overall surgical outcomes and recovery times.

Clinically, paraduodenal hernia accounts for 0.2-0.9% of intestinal obstruction [7]. They present with symptoms of intestinal obstruction, such as abdominal pain, nausea, and vomiting. However, Abukhalaf et al. (2019) reported that, in the majority of cases, it can be asymptomatic [9]. Symptoms persist for a chronic period, with intermittent abdominal pain. Due to the diagnostic challenges of identifying paraduodenal hernia as a cause of obstruction, it is essential to report such cases.

Our case aligns with previously reported cases in terms of clinical presentation, typically involving nonspecific abdominal pain and intermittent bowel obstruction. However, our management approach differed in utilizing laparoscopic repair instead of the traditional open repair methods commonly documented in earlier cases. The laparoscopic approach in our case offered advantages such as reduced postoperative pain, a shorter hospital stay, and a quicker recovery without compromising the efficacy of the repair. This minimally invasive technique provided enhanced visualization of the anatomical structures, facilitating a precise and effective surgical intervention.

In this case, we initially opted for a laparoscopic repair, which was converted to an exploratory laparotomy due to intraoperative findings. The laparoscopic approach was chosen for its benefits, including reduced morbidity and faster recovery [10]. During surgery, approximately 30-35 cm of jejunal loops herniating into the Landzert fossa were identified and reduced. The hernial sac was obliterated, and inter-mesenteric adhesiolysis was performed to alleviate the bowel obstruction. This comprehensive surgical approach effectively addressed the anatomical abnormalities and resolved the patient’s symptoms.

Managing a rare left paraduodenal hernia has significant clinical implications, particularly in terms of diagnosis and treatment. The nonspecific symptoms and subtle radiological findings often complicate early diagnosis. High-resolution CT scans with contrast are crucial for accurate identification. This case underscores the necessity of maintaining a high index of suspicion for internal hernias in patients with recurrent abdominal pain and intermittent bowel obstruction, even when initial imaging is inconclusive. Paraduodenal hernias can lead to severe complications, including bowel obstruction, ischemia, and perforation. While specific complication rates for paraduodenal hernias are limited due to the rarity of the condition, these potential outcomes underscore the critical importance of early diagnosis and prompt surgical intervention. Early management can significantly reduce the risk of life-threatening complications and improve patient outcomes [11,12].

This case provided several valuable lessons. Firstly, diagnostic pitfalls are common due to the nonspecific presentation and subtle imaging findings of left paraduodenal hernias. Enhanced imaging techniques, including high-resolution CT scans with enteric contrast, are recommended to improve diagnostic accuracy [5]. Secondly, the choice of surgical approach should be individualized based on the patient’s condition and anatomical considerations. The laparoscopic technique, while challenging, offers substantial benefits and should be considered when feasible [13]. Finally, early and accurate diagnosis, combined with prompt surgical intervention, is critical to managing these rare hernias effectively.

Left paraduodenal hernias are a rare medical condition, and improving their diagnosis and surgical outcomes requires a multi-pronged approach. Firstly, we recommend using high-resolution, contrast-enhanced CT scans as a routine part of the imaging protocol for patients with unexplained abdominal pain and suspected internal hernias. This will enhance our ability to identify these hernias accurately. Secondly, a multidisciplinary team approach is crucial. Collaboration between radiologists, gastroenterologists, and surgeons during diagnosis will ensure a comprehensive evaluation and well-planned treatment strategy. Finally, targeted training for surgeons in advanced laparoscopic techniques, particularly for rare hernia repairs like left paraduodenal hernias, is essential.

Establishing standardized guidelines for managing these rare hernias is necessary. These guidelines should encompass clear diagnostic criteria, recommend specific imaging protocols, outline indications for surgery and the appropriate surgical approach, and finally, detail postoperative care and follow-up protocols. This comprehensive strategy will lead to improved diagnostic accuracy and better surgical outcomes for patients with left paraduodenal hernias. 

Implementing such guidelines would promote consistent management practices, enhance patient outcomes, and contribute to ongoing research and data collection on rare hernias, ultimately improving the standard of care for these complex clinical entities.

## Conclusions

Left paraduodenal hernia (LPDH) is the most common type of congenital internal hernia but remains a rare entity that necessitates inclusion in the differential diagnosis for acute abdominal presentations. This case report highlights the diagnostic and therapeutic challenges associated with LPDH and intestinal malrotation in a 28-year-old male presenting with recurrent abdominal pain and intermittent bowel obstruction. Contrast-enhanced computed tomography (CT) showed altered relationships between the superior mesenteric artery (SMA) and vein (SMV), as well as a twisted distal ileal loop, after ultrasonography initially revealed the well-known "whirlpool sign." These findings emphasize the significance of a multifaceted diagnostic approach for accurate delineation of the underlying pathology. Timely surgical intervention with laparotomy effectively addressed the anatomical abnormalities and resolved the bowel obstruction. This case emphasizes the importance of early identification, prompt diagnosis, and surgery for LPDH with malrotation to improve patient outcomes.
